# Techniques for Precancerous Lesion Diagnosis

**DOI:** 10.1155/2011/326094

**Published:** 2011-01-13

**Authors:** Sarah Freygang Mendes, Grasieli de Oliveira Ramos, Elena Riet Correa Rivero, Filipe Modolo, Liliane Janete Grando, Maria Inês Meurer

**Affiliations:** ^1^Postgraduate Program of the Federal University of Santa Catarina, 88040-370 Florianopolis, SC, Brazil; ^2^Department of Pathology, Center of Health Sciences, Federal University of Santa Catarina, Trindade University Campus, 88040-370 Florianópolis, SC, Brazil

## Abstract

The development of the oral squamous cell carcinoma (OSCC) is a multistep process that requires the accumulation of multiple genetic alterations usually preceded by detectable mucosal changes, most often leukoplakias and erythroplakias. The clinical appearance of oral precancerous lesions and their degree of epithelium dysplasia suggests the malignization potential. Several techniques have been developed to improve the clinical and cytological diagnosis of oral precancerous lesions. The present paper reviews the main techniques used to improve premalignant lesion diagnosis.

## 1. Biopsy and Cytology

Oral cancer ranks as the sixth most common malignancy worldwide, 90% of which consists of squamous cell carcinoma [1–5]. Morbidity and mortality have not decreased over the past 50 years. Oral cancer early detection, mainly that of squamous cell carcinoma, is crucial to improve the patient's survival rate [1, 3–10]. The clinical diagnosis of oral precancerous lesions, leukoplakias and erythroplakias, is one of exclusion. The lesions to be excluded are those belonging to other conditions, such as lichen planus (acknowledging that it has a malignant potential itself), lupus erythematosus, leukoedema, white sponge nevus, and other lesions for which an etiology can be established, such as frictional keratosis, cheek/lip/tongue biting, contact lesions, and smoker's palate [11]. In many cases, a biopsy is mandatory so that such lesions can be discarded. Currently, histological criteria (dysplasia presence and degree) represent the gold standard in precancerous lesion risk evaluation [12]. 

## 2. Oral Exfoliative Cytology: Liquid-Based Preparations and Conventional Smears in Oral Lesions

Cytopathology is the microscopic study of cell samples collected from mucosal surfaces obtained by exfoliative cytology (via smears, scrapings, or lavage) or from internal sites via fine-needle aspiration [13]. Exfoliative cytology was first designed for cervical cancer cell early detection [14–18] and it has been primarily applied in oral medicine practice to detect early changes in oral mucosa related to malignancy. Furthermore, this exam has also been used in the diagnosis of certain types of oral lesions, most of which related to viral and fungal diseases [14]. 

Exfoliative cytology is performed with cytobrushes so as to obtain good-quality smear that includes cells from deeper layers of epithelium, especially of squamous intraepithelial lesions [19]. Cytological technique improvements that led to the development of a liquid-based preparation have renewed interest in the use of this approach as an auxiliary tool in oral lesion diagnosis [14]. 

According to Mehrotra et al. [19], sensitivity and specificity of conventional exfoliative cytology in carcinoma's suspected lesions, ranged between 76.8%–100%, and 88.9%–100%, respectively, in a review of 22 articles. In another paper, the cytological study of oral cavity's cells was shown to be suitable for routine application in population screening programs, for early analysis of suspect lesions, and for pre-and posttreatment monitoring of confirmed malignant lesions [16]. 

In liquid-based cytology, the cytobrush with the sample is transported in a vial containing preservative fluid which allows the immediate fixation of cells so that all the scraped material can be used, providing high cellularity slides dispersed in a thin and homogeneous layer on a clear background, thus facilitating abnormal cell identification [14, 17, 20]. These characteristics help to establish an early oral cancer diagnosis [17, 20–23]. The use of this technique has significantly reduced the number of unsatisfactory slides, diminishing false negative results and has increased sensitivity and specificity when compared to conventional cytology [14, 21]. According to Nanove [24], the sensitivity for this technique is 95,1%, and the specificity is 99%. In another study [19], it was showed the number of inadequate samples was reduced 8,8%. However, it requires more sophisticated laboratory equipment as well as a better-trained staff to handle, process, and analyze the samples properly [20].

The cells obtained from exfoliative cytology can be used for molecular analysis. Some molecular markers may provide additional information that will be useful in malignant lesion early diagnosis. The main markers used in cytological analysis are Ki-67 [16, page 53], [19, 22], DNA ploidy status (chromosomal pairing) [18, 25], epigenetic changes (hypermethylation of the promoter region), and genomic instability, such as loss of heterozygosity instability (LOH) [22, 26] and microsatellite (MSI) [22].

## 3. Cytomorphometry: Computer-Assisted Analysis Brush Biopsy

Cytomorphometry, computer-assisted analysis brush biopsy (Oral CDx Laboratories, Suffern, N.Y), is a method used in the analysis of cellular samples collected by brush biopsy, a disposable specialized circular plastic brush that collects transepithelial cellular samples composed of free cells and clusters [13, 19]. The clinician rubs or rotates the brush against the lesion until pinpoint bleeding is absorbed [27]. The samples are fixed onto a glass slide and sent to a laboratory where they are stained (via a modified Papanicolaou test), scanned, and analyzed microscopically by means of a computer-based imaging system that can rank cells on the basis of their degree of abnormal morphology [13]. The analytical results and representative examples are then referred to a pathologist [19]. Results are reported as “negative” or “benign,” “positive” or “atypical.” Abnormal diagnoses have included “positive” (defined as definitive cellular evidence of epithelial dysplasia or carcinoma) and “atypical” (defined as abnormal epithelial changes of uncertain diagnostic significance) results [13]. 

After the automated analysis, the pathologist can recommend the clinical practitioner to follow further procedures (clinical control, repeated brush biopsy, surgical biopsy, etc.) [19]. Several papers have been written on this technique [19, 28–37], but few have evaluated its performance in the prevention of oral cancer [13, 30–32, 34, 38–40]. These articles have reported sensitivity values that ranged from 88% [39] to 100% [32] and 25% [38] to 96% specificity [39]. This test has been chosen to assess lesions the practitioner might not investigate further and is not recommended for the assessment of clinically suspicious lesions for which the practitioner would normally perform conventional biopsy [13, 17]. 

Oral brush biopsy, as a noninvasive diagnostic method, can be useful for oral mucosal lesion early detection. The occurrence of positive findings, or lesion progression despite negative findings, signals that the patient needs to be referred to a specialized clinic where a surgical biopsy should be performed, followed by histopathologycal analysis. Histopathology remains the gold standard for the definitive diagnosis of oral malignant lesions [36, 41].

## 4. Clinical Tissue Staining Technique

### 4.1. Vital Iodine Stain

Vital iodine stain (3% Lugol solution) can be used prior to biopsy and resection and is useful in the determination of the best incision area. This technique has been used in upper gastrointestinal tract endoscopy routine, as well as in cervix examination and in esophageal cancer [42]. Its principle is based on the binding of iodine to glycogen granules in the cytoplasm, resulting in a black-brown tissue color. In cancer cells, where the glycolysis is elevated [42], this method results in unstained areas whereas the normal mucosa is stained [42]. In a study with 54 patients [42], with oral squamous cell carcinoma or oral potentially malignant lesions, where the authors made surgical margins of 5–8 mm from the border of the stained lesion with vital iodine stain, it was shown that 98,1% has no recurrence after a median followup of 15 months.

### 4.2. Toluidine Blue Staining (TBlue Staining)

Toluidine Blue (also known as tolonium chloride) is a vital metachromatic dye of the thiazine group that has been effectively used in nuclear staining because of its binding to DNA nucleus acid [2, 3, 7]. It has been used for decades as an aid in epithelium dysplasia identification [2, 10] and appears to improve precancerous lesion visualization by showing high-risk areas (areas of high cell proliferation), therefore guiding biopsy [2, 3, 7, 9, 10]. However, most studies have had problems with the absence of randomized control care and methodology [2, 3, 7, 9, 10]. The proceeding starts with a topical application of TBlue on the lesion with the aid of a swab or cotton applicator, and the more intense TBlue staining areas should be the ones elected to be biopsied. TBlue seems to be highly sensitive but has low specificity, since it also stains benign and common lesions which involve inflammation [2, 7]. TBlue is an easy and cheap technique, causes no harm to the patient, and may help to perform a careful clinical examination [3, 7]. False negative staining is very rarely observed in squamous cell carcinoma, but inflammatory lesions can contribute to false positive outcomes [7]. Some works [7, 10] have shown the sensitivity and specificity vary from 38%–98% and 9%–93%, respectively.

## 5. Chemiluminescence Technique

### 5.1. Chemiluminescence Light

The chemiluminescence technique (ViziLite (Zila Pharmaceuticals, Phoenix, Arizona)) is an exam that was approved in 2002 in the USA. It serves the purpose of improving the identification, visualization, and monitoring of oral precancerous lesions [1, 4, 8, 10, 43], and consists of the emission of light from a chemical reaction between hydrogen peroxide and acetylsalicylic acid inside a capsule light stick [4, 8, 44]. The use of a 1% acetic acid solution for washing and cleaning the oral mucosa for about 1 minute before chemiluminescence light is recommended. The action of the stick holds good for approximately 10 minutes [1, 3, 4, 44]. This reaction emits a blue/white light (430–580 nm) whose principle is based on the reflective properties of tissues that present cellular alterations such as a higher nuclear/cytoplasmatic rate. The “acetowhite” lesion is more defined and sharper, whereas the normal tissue is dark [1–4, 8–10, 44].

This seems to be an easy, safe and noninvasive system capable of helping the dentist to better visualize lesions, as well as its edges [3, 8, 44]. Another point to consider is that the lesion seems to be bigger under chemiluminescence light [44]. One disadvantage is that this system is expensive and a stick is used for each patient. Furthermore, chemiluminescence light seems to be nonspecific as it does not identify the lesion etiology—whether inflammatory, neoplastic benign, or neoplastic malign—and this could lead to unnecessary biopsies [2, 8, 44]. 

This system is useful for clinical examination, inasmuch as it improves the lesion visualization [9], especially when used in association with the Toluidine Blue solution to mark the lesions to posterior biopsy [2, 8]. It is known that acetic acid wash can provide a more accurate diagnosis than chemiluminescence light [2]. 

The ViziLite tool enhance intraoral visualization of white lesions, however it is not able to discriminate between keratotic, inflammatory, malignant, or potentially malignant oral mucosal white lesions [8, 9, 45]. The main advantage of this technique is that it significantly improves the sharpness of the lesions' margins [8, 9, 45].

## 6. Light Emission Technique

The Light Emission Technique (Microlux DL (AdDent, Danbury, Conn.)) seems to operate on a principle of light emission similar to that of chemiluminescence light and helps to sharpen the lesion edges as well as to improve visualization [2, 43]. In this method, the patient first needs to have a 1% acetic acid mouthwash, and then a battery-powered light source is used. An advantage of this system is that it is reusable [2, 8, 10, 43]. Another similar system uses a Led (Orascoptic DK (Orascoptic, a Kerr Company, Middleton, Wis.)) with a rechargeable battery to screen the oral mucosa and claims to improve visualization [10]. In a study [43] to assess the efficacy of acetic acid mouthwash and diffused light illumination (Microlux/DL), as a diagnostic tool in the visualization of oral mucosal potentially malignant lesions, Microlux/DL showed a sensitivity of 77.8% and a specificity of 70.7%, with a positive predictive value of 36.8%. According to the authors [43], Microlux/DL was able to enhance lesion visibility; however, it is a poor discriminator for inflammatory, traumatic, and malignant lesions.

The Narrow-emission tissue fluorescence (VELscope (LED Dental Inc. White Rock, British Columbia, Canada)) technique involves tissue exposure to different wavelengths (400 to 460 nm) in order to observe differences between normal and abnormal mucosa [2, 46]. This system involves the cell answer (autofluorescence due to cellular fluorophores) after excitation [2–4, 10]. The abnormal tissue has a different fluorophore concentration that results in changes in color [4]. This method uses a small optic fiber and consequently does not cover the entire mouth, so it is employed only for isolated lesions [2], lesion edge, and cancerization field determination [4]. While the normal mucosa glows and emits color (pale green), the abnormal mucosa shows decreased levels of fluorescence and acquires a dark magenta, brown, or black color, as it absorbs fluorescence [2, 4, 6, 47]. This technique seems to be helpful in lesion detection, but it is useless in the differentiation of malignant from benign lesions [2, 3, 6]. Despite its applicability, the system is expensive, and color interpretation is difficult, which could lead to a erroneous diagnosis [6]. Some studies in the literature [4, 6] referred this technique as having sensitivity values from 97% to 98% and specificity from 94% to 100%.

Multispectral fluorescence and reflectance (Identafi 3000 (Trimira, Houston, Texas)) is a new technique based on the tissue fluorescence principle [3, 46] which uses three types of lights: white, violet (405 nm), and amber (560 nm) [46]. According to the manufacturer, white and violet lights use the same principle as tissue reflex and fluorescence, while amber light improves vascular architecture visualization in normal and abnormal tissue [48]. Normal tissue appears defined, while the abnormal tissue has a diffuse vasculature [48]. According to the manufacturer, it is reusable [48]. This method has not been studied yet.

## 7. Conclusions

In this paper we approached different techniques which may be useful in the diagnosis of precancerous lesions. It is shown their applications and limitations. However, many possibilities are available, and it is concluded that the most reliable method of diagnosis is still the biopsy followed by histopathological examination.

## References

[1] Oh ES, Laskin DM (2007). Efficacy of the ViziLite system in the identification of oral lesions. *Journal of Oral and Maxillofacial Surgery*.

[2] Lingen MW, Kalmar JR, Karrison T, Speight PM (2008). Critical evaluation of diagnostic aids for the detection of oral cancer. *Oral Oncology*.

[3] Rhodus NL (2009). Oral cancer and precancer: improving outcomes. *Compendium of Continuing Education in Dentistry*.

[4] Trullenque-Eriksson A, Muñoz-Corcuera M, Campo-Trapero J, Cano-Sánchez J, Bascones-Martínez A (2009). Analysis of new diagnostic methods in suspicious lesions of the oral mucosa. *Medicina Oral, Patologia Oral y Cirugia Bucal*.

[5] Kujan O, Glenny AM, Oliver RJ, Thakker N, Sloan P (2006). Evaluation of screening strategies for improving oral cancer mortality: a Cochrane systematic review. *Cochrane Database of Systematic Reviews*.

[6] Balevi B (2007). Evidence-based decision making: should the general dentist adopt the use of the VELscope for routine screening for oral cancer?. *Journal of the Canadian Dental Association*.

[7] Epstein JB, Güneri P (2009). The adjunctive role of toluidine blue in detection of oral premalignant and malignant lesions. *Current Opinion in Otolaryngology and Head and Neck Surgery*.

[8] Farah CS, McCullough MJ (2007). A pilot case control study on the efficacy of acetic acid wash and chemiluminescent illumination (ViziLite) in the visualisation of oral mucosal white lesions. *Oral Oncology*.

[9] Epstein JB, Silverman S, Epstein JD, Lonky SA, Bride MA (2008). Analysis of oral lesion biopsies identified and evaluated by visual examination, chemiluminescence and toluidine blue. *Oral Oncology*.

[10] Patton LL, Epstein JB, Kerr AR (2008). Systematic review of the literature examination and lesion diagnosis: adjunctive techniques for oral cancer. *Journal of the American Dental Association*.

[11] Reibel J (2003). Prognosis of oral pre-malignant lesions: significance of clinical, histopathological, and molecular biological characteristics. *Critical Reviews in Oral Biology and Medicine*.

[12] Schepman KP, Van der Waal I (1995). A proposal for a classification and staging system for oral leukoplakia: a preliminary study. *European Journal of Cancer Part B*.

[13] Patton LL, Epstein JB, Kerr AR (2008). Adjunctive techniques for oral cancer examination and lesion diagnosis a systematic review of the literature. *Journal of the American Dental Association*.

[14] Hunter KD, Parkinson EK, Harrison PR (2005). Profiling early head and neck cancer. *Nature Reviews Cancer*.

[15] Kao SY, Chen YW, Chang KW, Liu TY (2009). Detection and screening of oral cancer and pre-cancerous lesions. *Journal of the Chinese Medical Association*.

[16] Mehrotra R, Gupta A, Singh M, Ibrahim R (2006). Application of cytology and molecular biology in diagnosing premalignant or malignant oral lesions. *Molecular Cancer*.

[17] Mehrotra R, Singh MK, Pandya S, Singh M (2008). The use of an oral brush biopsy without computer-assisted analysis in the evaluation of oral lesions: a study of 94 patients. *Oral Surgery, Oral Medicine, Oral Pathology, Oral Radiology and Endodontology*.

[18] Remmerbach TW, Weidenbach H, Hemprich A, Böcking A (2003). Earliest detection of oral cancer using non-invasive brush biopsy including DNA-image-cytometry: report on four cases. *Analytical Cellular Pathology*.

[19] Mehrotra R, Hullmann M, Smeets R, Reichert TE, Driemel O (2009). Oral cytology revisited. *Journal of Oral Pathology and Medicine*.

[20] Hayama FH, Motta ACF, De Padua G Silva A, Migliari DA (2005). Liquid-based preparations versus conventional cytology: specimen adequacy and diagnostic agreement in oral lesions. *Medicina Oral, Patologia Oral y Cirugia Bucal*.

[21] Navone R, Burlo P, Pich A (2006). The impact of liquid-based oral cytology on the diagnosis of oral squamous dysplasia and carcinoma. *Cytopathology*.

[22] Acha A, Ruesga MT, Rodríguez MJ, Martínez De Pancorbo MA, Aguirre JM (2005). Applications of the oral scraped (exfoliative) cytology in oral cancer and precancer. *Medicina Oral, Patologia Oral y Cirugia Bucal*.

[23] Spafford MF, Koch WM, Reed AL (2001). Detection of head and neck squamous cell carcinoma among exfoliated oral mucosal cells by microsatellite analysis. *Clinical Cancer Research*.

[24] Navone R (2009). Cytology of the oral cavity: a re-evaluation. *Pathologica*.

[25] Maraki D, Becker J, Boecking A (2004). Cytologic and DNA-cytometric very early diagnosis of oral cancer. *Journal of Oral Pathology and Medicine*.

[26] Zhang L, Rosin MP (2001). Loss of heterozygosity: a potential tool in management of oral premalignant lesions?. *Journal of Oral Pathology and Medicine*.

[27] Rethman MP, Carpenter W, Cohen EEW (2010). Evidence-based clinical recommendations regarding screening for oral squamous cell carcinomas. *Journal of the American Dental Association*.

[28] Eisen D (2009). The role of the OralCdx brush test in preventing oral cancer. *Dental Assistant*.

[29] Kosicki DM, Riva C, Pajarola GF, Burkhardt A, Grätz KW (2007). OralCDx brush biopsy—a tool for early diagnosis of oral squamous cell carcinoma. *Schweizer Monatsschrift für Zahnmedizin*.

[30] Poate TWJ, Buchanan JAG, Hodgson TA (2004). An audit of the efficacy of the oral brush biopsy technique in a specialist Oral Medicine unit. *Oral Oncology*.

[31] Scheifele C, Schmidt-Westhausen AM, Dietrich T, Reichart PA (2004). The sensitivity and specificity of the OralCDx technique: evaluation of 103 cases. *Oral Oncology*.

[32] Sciubba JJ (1999). Improving detection of precancerous and cancerous oral lesions: computer-assisted analysis of the oral brush biopsy. *Journal of the American Dental Association*.

[33] Trullenque-Eriksson A, Muñoz-Corcuera M, Campo-Trapero J, Cano-Sánchez J, Bascones-Martínez A (2009). Analysis of new diagnostic methods in suspicious lesions of the oral mucosa. *Medicina Oral, Patologia Oral y Cirugia Bucal*.

[34] Delavarian Z, Mohtasham N, Mosannen-Mozaffari P, Pakfetrat A, Shakeri M-T, Ghafoorian-Maddah R (2010). Evaluation of the diagnostic value of a modified liquid-based cytology using OralCDx ® brush in early detection of oral potentially malignant lesions and oral cancer. *Medicina Oral, Patologia Oral y Cirugia Bucal*.

[35] Bhoopathi V, Kabani S, Mascarenhas AK (2009). Low positive predictive value of the oral brush biopsy in detecting dysplastic oral lesions. *Cancer*.

[36] Hohlweg-Majert B, Deppe H, Metzger MC (2009). Sensitivity and specificity of oral brush biopsy. *Cancer Investigation*.

[37] Christian DC (2002). Computer-assisted analysis of oral brush biopsies at an oral cancer screening program. *Journal of the American Dental Association*.

[38] Svirsky JA, Burns JC, Carpenter WM (2002). Comparison of computer-assisted brush biopsy results with follow up scalpel biopsy and histology. *General Dentistry*.

[39] Driemel O, Dahse R, Berndt A (2007). High-molecular tenascin-C as an indicator of atypical cells in oral brush biopsies. *Clinical Oral Investigations*.

[40] Hirshberg A, Yarom N, Amariglio N (2007). Detection of non-diploid cells in premalignant and malignant oral lesions using combined morphological and FISH analysis—a new method for early detection of suspicious oral lesions. *Cancer Letters*.

[41] Hullmann M, Reichert TE, Dahse R (2007). Oral cytology: historical development, current status, and perspectives. *Mund-, Kiefer- und Gesichtschirurgie*.

[42] Maeda K, Suzuki T, Ooyama Y (2010). Colorimetric analysis of unstained lesions surrounding oral squamous cell carcinomas and oral potentially malignant disorders using iodine. *International Journal of Oral and Maxillofacial Surgery*.

[43] McIntosh L, McCullough MJ, Farah CS (2009). The assessment of diffused light illumination and acetic acid rinse (Microlux/DL*™*) in the visualisation of oral mucosal lesions. *Oral Oncology*.

[44] Ram S, Siar CH (2005). Chemiluminescence as a diagnostic aid in the detection of oral cancer and potentially malignant epithelial lesions. *International Journal of Oral and Maxillofacial Surgery*.

[45] Ram S, Siar CH (2005). Chemiluminescence as a diagnostic aid in the detection of oral cancer and potentially malignant epithelial lesions. *International Journal of Oral and Maxillofacial Surgery*.

[46] Vigneswaran N, Koh S, Gillenwater A (2009). Incidental detection of an occult oral malignancy with autofluorescence imaging: a case report. *Head & Neck Oncology*.

[47] Paulis M (2009). The influence of patient education by the dental hygienist: acceptance of the fluorescence oral cancer exam. *Journal of Dental Hygiene*.

[48] Trimera How it works?. http://www.trimira.net/identafi.

